# A Turbidity-Compensation Method for Nitrate Measurement Based on Ultraviolet Difference Spectroscopy

**DOI:** 10.3390/molecules28010250

**Published:** 2022-12-28

**Authors:** Jing Dong, Junwu Tang, Guojun Wu, Ruizhuo Li

**Affiliations:** 1 Xi’an Institute of Optics and Precision Mechanics, Chinese Academy of Sciences, Xi’an 710119, China; 2 University of Chinese Academy of Sciences, Beijing 100049, China; 3 Pilot National Laboratory for Marine Science and Technology (Qingdao), Qingdao 266237, China; 4 Faculty of Information Science and Engineering, Ocean University of China, Qingdao 266100, China

**Keywords:** nitrate, ultraviolet spectroscopy, turbidity compensation, difference spectrum, partial least squares

## Abstract

To solve the problem that turbidity in water has a significant effect on the spectra of nitrate and reduces the accuracy of nitrate detection, a turbidity-compensation method for nitrate measurement based on ultraviolet difference spectra is proposed. The effect of turbidity on the absorption spectra of nitrate was studied by using the difference spectra of the mixed solution and a nitrate solution. The results showed that the same turbidity had different effects on the absorbance of different concentrations of nitrate. The change in absorbance due to turbidity decreased with an increase in the nitrate concentration at wavelengths from 200 nm to 230 nm, although this change was constant when the wavelength was greater than 230 nm. On the basis of this characteristic, we combined the residual sum of squares (RSS) and interval partial least squares (iPLS) to select wavelengths of 230–240 nm as the optimal modeling interval. Furthermore, the turbidity-compensation model was established by the linear fitting of the difference spectra of various levels of turbidity. The absorption spectra of the nitrate were extracted by subtracting the turbidity-compensation curve from the original spectra of the water samples, and the nitrate concentration was calculated by using a partial least squares (PLS)-based nitrate-prediction model. The experimental results showed that the average relative error of the nitrate predictions was reduced by 50.33% to 1.33% by the proposed turbidity-compensation method. This indicated that this method can better correct the deviation in nitrate’s absorbance caused by turbidity and improve the accuracy of nitrate predictions.

## 1. Introduction

Nitrate is a key phytoplankton nutrient. The concentrations of nitrate in seawater can affect marine primary productivity and regulate the structure of the phytoplankton community [1]. The accurate monitoring and analysis of nitrate concentrations are essential for understanding biogeochemical cycles and preventing ecological imbalances caused by high nitrate concentrations [2].

Traditionally, the laboratory chemical method has been used to determine nitrate levels [3]. Water samples are collected by a survey ship and transported to a laboratory for analysis. However, this method cannot capture the continuous temporal and spatial variation in nitrate. Through a combination of flow-injection analysis (FIA) [4] and/or microfluidic technology [5], nitrate sensors based on wet-chemical methods can be used for in situ nitrate detection. However, the problems related to preserving the chemical reagent and waste discharge limit the wide application of these sensors [6].

Ultraviolet spectroscopy is a popular method used for determining concentrations of nitrate with the significant advantages of simplicity, rapidity, high accuracy, and lack of reagent. In addition, ultraviolet spectroscopy has also been increasingly used to measure other water-quality parameters [7,8,9]. Johnson and Coletti [10] developed an in situ ultraviolet spectrophotometer (ISUS) for the direct high-resolution measurement of nitrate, bromide, and bisulfide. Langergraber et al. [11] presented a submersible UV-vis spectrometer to monitor nitrate, chemical oxygen demand (COD), and total suspended solids (TSS). However, matrix effects can seriously interfere with the detection of nitrates in natural water, and one of the most critical interfering factors is turbidity caused by suspended particulate matter [12]. The scattering of light by suspended particles reduces the amount of light reaching the detector, which changes the magnitude and peak position of the nitrate’s absorption spectrum, thereby affecting the calculation of the nitrate concentration [13]. Therefore, eliminating the interference of turbidity is necessary.

Various turbidity-compensation methods have been proposed to reduce or eliminate the interference of turbidity. There are two main strategies. One is to establish the relationship between before and after turbidity correction by the mapping function of the model to recover the pure absorbance spectra of the water-quality parameter for analysis. Examples include the multiplicative scatter correction (MSC) algorithm [9,11], the orthogonal signal correction (OSC) algorithm [14], and deep-learning methods [15]. These methods can suppress the interference of turbidity to a certain degree but cannot fundamentally eliminate its effect. The accuracy of the model depends on the size of the training sample, and it is often necessary to retrain the model in the face of new water bodies. Another turbidity-compensation method is to subtract the absorbance caused by turbidity from the original spectrum based on a single wavelength [16,17] or multiple wavelengths [18,19,20,21]. Because the multi-wavelength method provides more information than single-wavelength methods, it is more extensively used. On the basis of Mie’s scattering theory, Chen et al. effectively predicted the extinction spectrum induced by particulate matter and managed to compensate for turbidity [18]. However, it is difficult to accurately estimate the negative refractive index of suspended particulate matter in natural water. Chen et al. proposed a compensation-curve method to correct the absorption spectrum of nitrate. The compensation-curve model was established by a mixed solution of 2 mg/L nitrate and 5–50 NTU turbidity [19]. Li et al. reconstructed an influence matrix of the turbidity-absorption spectra based on a compressed sensing algorithm and used it to eliminate the interference of turbidity in COD detection [20]. Cai et al. calculated a corrected-absorbance spectral curve by introducing a coefficient K_N_, which describes the degree to which the absorption spectrum of a turbid mixed COD solution deviates from the superposition law [21]. These kinds of turbidity-interference studies have been conducted with constant nitrate concentrations, varying the turbidity to examine its impact on the nitrate’s absorption spectrum. However, this ignores a problem: whether the same level of turbidity has the same effect on varying nitrate concentrations. If the turbidity-compensation model is established in this way, it inevitably affects the accuracy with which other nitrate concentrations not included in the modeled sample set are detected.

In this study, the effect of turbidity on the absorption spectra of nitrate at different wavelengths was studied. Moreover, a turbidity-compensation method based on difference spectra combined with linear fitting is proposed. The turbidity was determined by the spectral area, and the concentration of nitrate was finally determined using a multivariate-calibration diffmodel. The results demonstrated that the proposed method can significantly enhance the accuracy of nitrate predictions.

## 2. Results and Discussion

### 2.1. Analysis of the Interference of Turbidity

**Figure 1:**
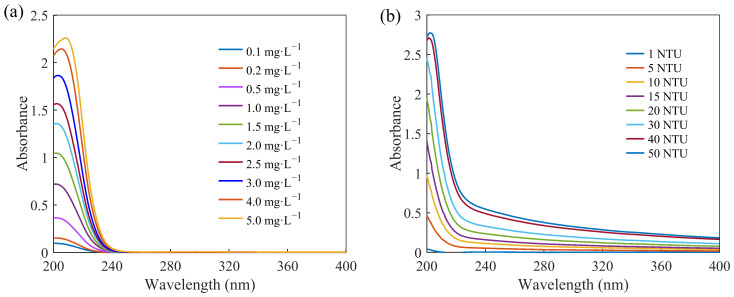
(**a**) Absorption spectra of solutions with different nitrate concentrations. (**b**) Absorption spectra of standard solutions with different levels of turbidity.

The absorption spectra of the nitrate and turbidity solutions were measured, and the curves are shown in Figure 1a,b, respectively. The main spectral-absorption range of the nitrate was 200–250 nm, and the absorbance of nitrate was almost zero after 250 nm. The absorption spectra of the turbidity solutions covered the entire ultraviolet band, which could cause spectral cross-sensitivity and interfere with the absorption spectra of the nitrate solutions.

We prepared 15 groups of mixed solutions of nitrate and turbidity according to the sample concentrations and collected their absorption spectra. The spectra of the nitrate solutions were subtracted from the spectra of the mixed solutions to obtain their difference spectra.

**Figure 2:**
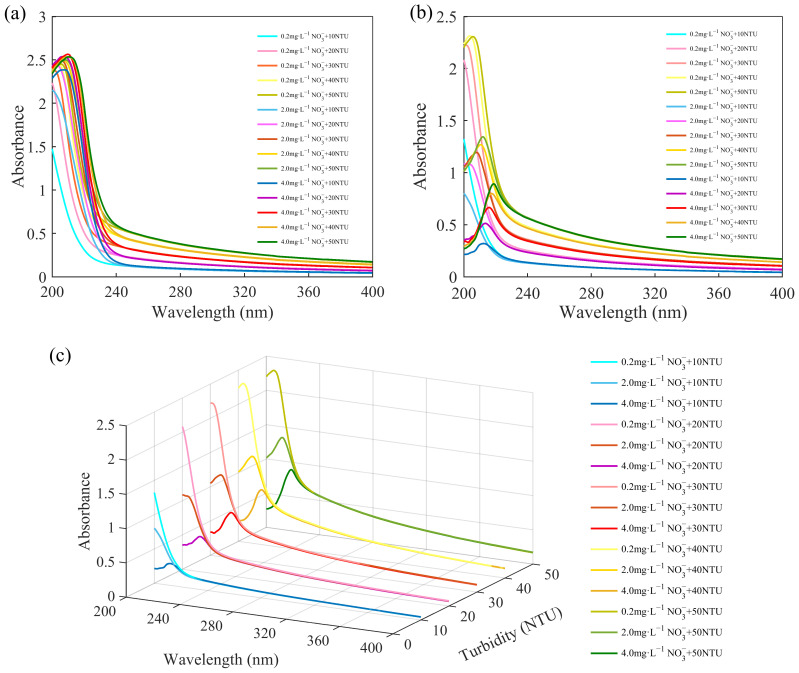
(**a**) Spectra of mixed solutions. (**b**) Difference spectra between the mixed solutions and standard nitrate solutions. (**c**) Difference spectra in three-dimensional coordinates.

The spectra of the mixed solutions and the difference spectra are shown in Figure 2. The spectra of the mixed solutions in Figure 2a were subjected to strong interference by the turbidity. As can be seen in Figure 2b,c, the amount of change in the absorbance caused by turbidity at different wavelengths varied. When the concentration of the nitrate was constant, the absorbance of the difference spectra increased with the increase in turbidity. However, when the turbidity was constant, the effect of the turbidity at different wavelengths changed as the nitrate concentration changed. At wavelengths of less than about 230 nm, the absorbance of the solutions with the same level of turbidity and different nitrate concentrations decreased with the increase in the nitrate concentration. At wavelengths greater than about 230 nm, the difference spectra almost overlapped. This means that the same turbidity had the same effect on the absorbance of the nitrate in this wavelength range.

Changes in absorbance are related to the effect of turbid particles in the solution on the nitrate molecules. The difference spectra reflect this effect, which includes two parts: one is the increase in absorbance caused by the absorption and scattering of suspended particles in turbid solutions, and the other is that suspended particles break the coplanar nature of nitrate molecules, causing steric hindrance and destroying the conjugate system, leading to a decrease in the absorbance of nitrate [19]. The superposition of these two parts is the change in the absorbance of nitrate caused by the inference of turbidity. Therefore, the superposition of the absorbance of turbidity solutions and nitrate solutions is usually greater than the absorbance of the mixed solution. Similar results were obtained in the research by Chen et al. [19].

Furthermore, the effect of turbidity was greatest at the wavelength of the absorption peak and decreased with the decrease in the absorbance. This issue was discussed by Hu et al., who concluded that the effect of turbidity was greatest at the central wavelength of the energy leap and became smaller with the decrease in the probability of energy leaps [13]. Therefore, the selection of the correct wavelength is necessary to account for the different effects of turbidity at different wavelengths.

We calculated the correlation coefficient between the difference spectra and the turbidity. In the band in which the difference spectra overlapped, the correlation coefficient was above 0.99. This demonstrates that the effect of turbidity on the absorbance of nitrate in this spectral band is proportional to the turbidity.

Based on this characteristic, a turbidity-compensation model was established by linear regression in a spectral interval. The effect of the turbidity on the nitrate was constant in this interval. Therefore, the mixed solutions with random levels of turbidity and random concentrations of nitrate were corrected accurately.

### 2.2. Interval Selection

The optimal interval is both the modeling interval of the turbidity-compensation model and the nitrate prediction model. Therefore, a suitable spectral interval for the turbidity-compensation model was chosen according to the characteristic effects of turbidity on the absorption spectra of nitrate. The interval was further optimized, and the interval with the highest accuracy in the nitrate-prediction model was taken as the final optimal interval.

It was shown in the previous section that the interval that is most suitable for establishing the turbidity-compensation model is the interval in which the difference spectra overlap. To extract the overlapping bands in the difference spectra, the difference spectra for a nitrate content of 0.2 mg/L were utilized as the reference spectra, and the evaluation method was the residual sum of squares (RSS) of the other difference spectra and the reference spectra. When the RSS was less than a specific threshold, this indicated that the difference in the spectra in this range was quite small, and the spectral curves essentially overlapped. The method of calculation is as follows

(1) $$\mathrm{RSS}=\overset{m}{\underset{i=1}{\sum }}\overset{n}{\underset{j=1}{\sum }}{\left(y_{ij}-y_{i0}\right)}^{2}$$

where $y_{\mathrm{ij}}$ is the jth difference spectrum at the ith level of turbidity, $y_{i0}$ is the reference spectrum at the ith level of turbidity, n is the number of difference spectra apart from the reference spectrum, and m is the number of different turbidity levels.

**Figure 3:**
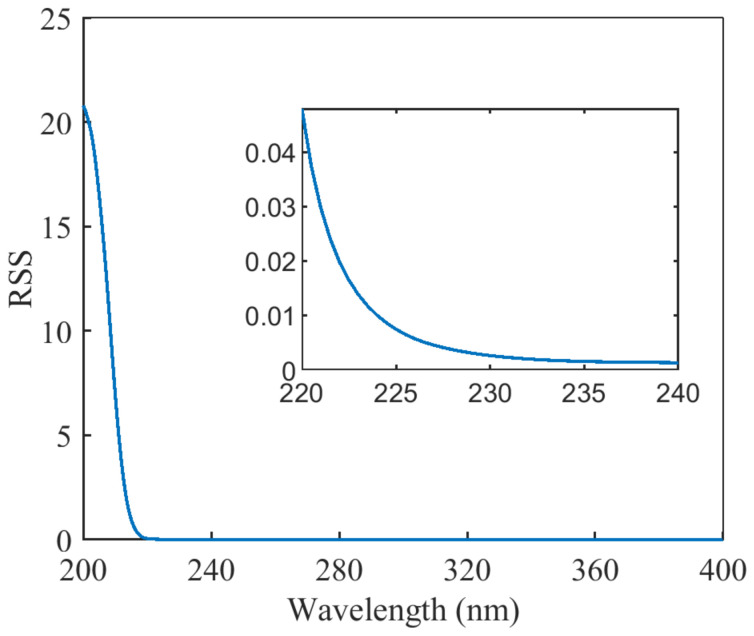
RSS at different wavelengths.

Figure 3 shows the result of the RSS at different wavelengths. The RSS was less than 0.003 at wavelengths greater than 230 nm. Therefore, when this band was selected for turbidity-compensation modeling, the change in the absorbance of the mixed solution was only related to the turbidity and not to the nitrate concentration.

The wavelength range of 230–400 nm is still quite wide for establishing a nitrate-prediction model. This may include uninformative wavelengths, which reduce the accuracy of the prediction and increase the data-processing time.

**Figure 4:**
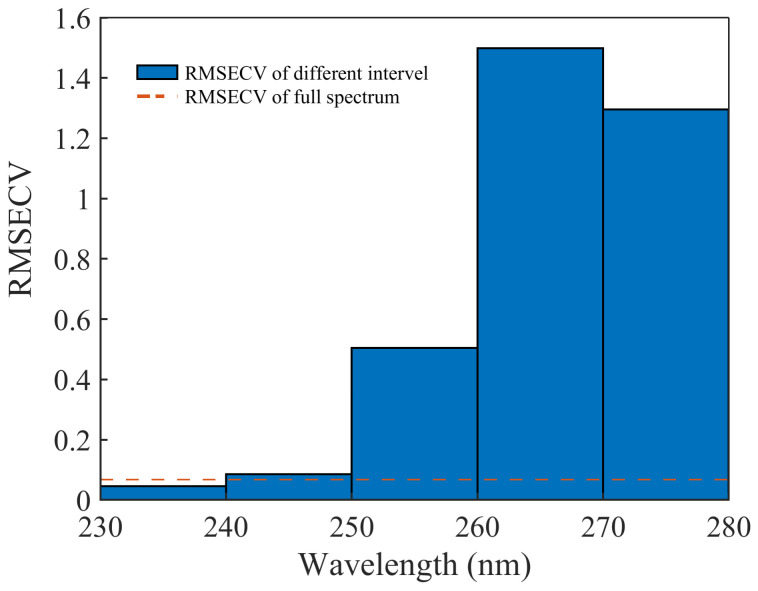
Interval optimization by interval-partial-least-squares method.

The interval partial least squares (iPLS) method proposed by Norgaard et al. was used to optimize the wavelength range. The spectrum was divided into different intervals. The RMSECV for each interval was calculated, and the interval with the lowest RMSECV was chosen as the optimal wavelength interval. We divided the 230–400 nm wavelength band in the spectra data of the standard nitrate solutions into 17 intervals: 230–240, 240–250, …, 390–400 nm. The wavelength range with the lowest RMSECV was chosen by iPLS. For the range of 230–240 nm, the RMSECV value was the lowest, which was also lower than that of the model with the full spectral range. The result is shown in Figure 4 (which plots the first five intervals). Therefore, the modeling interval was finally determined to be 230–240 nm.

### 2.3. Establishment of the Turbidity-Compensation Model

**Figure 5:**
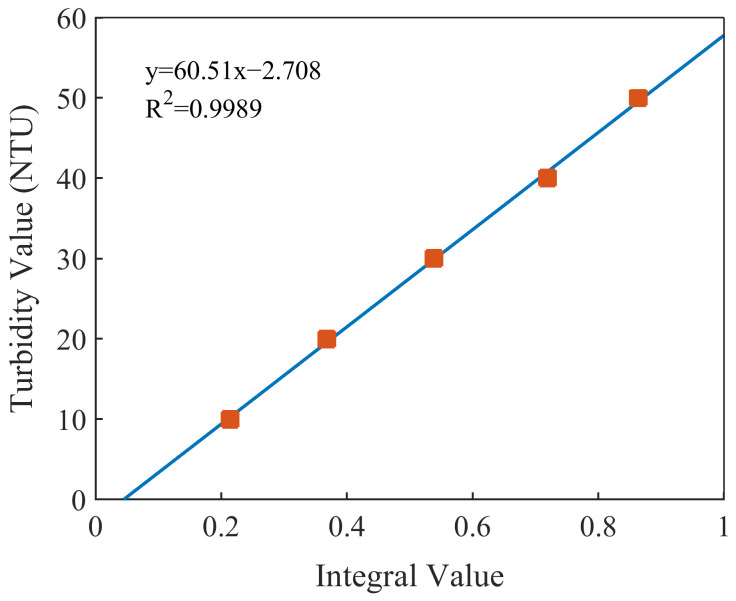
Fitting relationship between the integral values and the turbidity values.

The turbidity-prediction model was established by linear regression combined with the spectral area. According to the spectral characteristics of the nitrate and the turbidity, in the wavelength range of 250–400 nm, the absorbance of the nitrate solutions was almost zero and the absorption spectra of the mixed solutions were almost entirely attributable to the turbidity. In addition, the correlation coefficients of the absorbance and the turbidity at each wavelength in this band were greater than 0.9. The selection of this band can also eliminate the problem of the susceptibility of single-wavelength regression modeling to interference. The 15 spectral curves of the mixed solutions were divided into five groups, and those with the same level of turbidity were grouped together. Five spectral curves were obtained by averaging each group. We then integrated the five spectral curves between 250 and 400 nm and obtained their spectral areas. The integral values and turbidity values were used to build a turbidity-prediction model. The results of the modeling are shown in Figure 5. The linear-regression equation was y = 60.51x − 2.708, and the R^2^ was 0.9989.

The turbidity-compensation model was established by using the difference spectra at 230–240 nm. The mean values of the difference spectra with the same level of turbidity were calculated to obtain the reference compensation curve. The compensation curve of any turbidity value can be calculated by linear fitting, as described in Section 3.4. The regression parameters a_i_ (i = 1, 2,…, λ) and b_i_ (i = 1, 2,…, λ) for each wavelength were calculated as shown in Table 1. After calculating the turbidity of the unknown mixed solution using this turbidity-prediction model, we substituted the turbidity value into Formula (5) in Section 3.4 to obtain the turbidity-compensation curve. The spectra of nitrate can be extracted by subtracting the compensation curve from the mixed solution’s spectra.

### 2.4. Establishment of the Nitrate-Prediction Model

**Figure 6:**
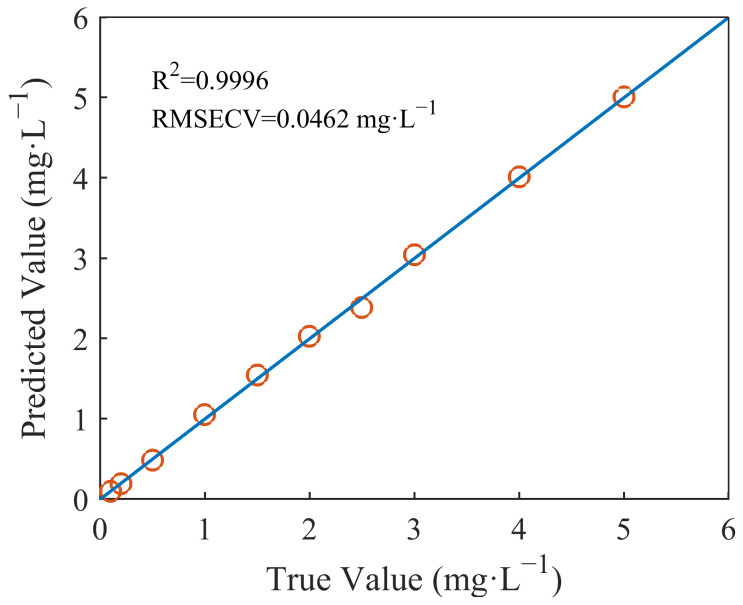
Relationship between the true values and the values of nitrate predicted by PLS.

The nitrate-prediction model was established by the PLS method. The nitrate samples were used as a calibration set. We used leave-one-out cross-validation to verify the model and RMSECV to evaluate the model’s accuracy. The model showed good accuracy. The R^2^ of the predicted values and the true values was 0.9996, and the RMSECV was 0.0462 mg/L. The plot of the predicted concentrations versus the actual concentrations is shown in Figure 6.

### 2.5. Experiment with the Random Mixed-Solution Samples

To verify the turbidity-compensation method proposed, we prepared five mixed solutions with random concentrations of nitrate and levels of turbidity.

**Figure 7:**
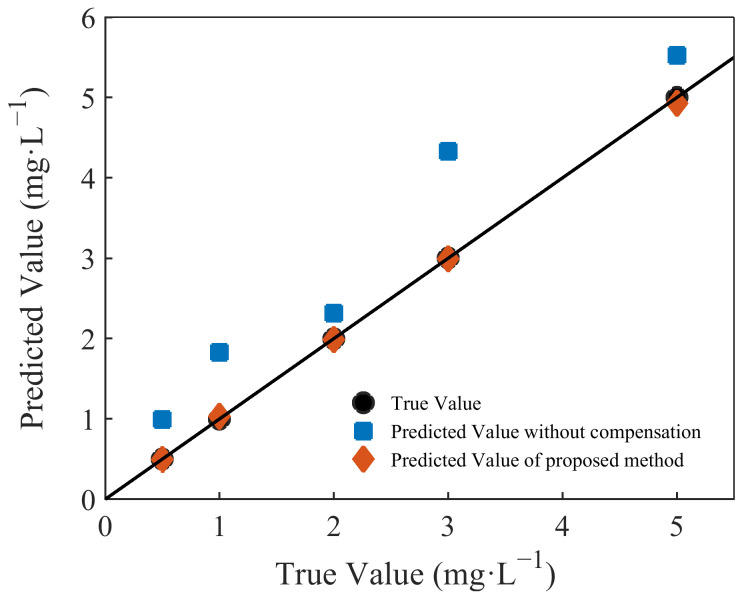
Comparison of the results of uncompensated and compensated predictions.

The absorption spectra of the five groups of mixed solutions were collected. The spectra of the mixed solutions were corrected by the turbidity-compensation model, after which the nitrate concentrations were calculated by substitution in the nitrate-prediction model. The results of the predictions before and after compensation are plotted in Figure 7, and the relative errors, RMSEP, and R^2^ values before and after compensation are shown in Table 2.

It can be seen that the results of the uncompensated prediction were quite different from the true values. As the turbidity of the mixed solution increased, the error in the prediction of the nitrate concentration became increasingly significant. After compensation, the predicted values were generally consistent with the true values. The average relative error decreased from 50.33% to 1.33%, and the RMSEP value was very small. The accuracy with which the nitrate was predicted was significantly improved after compensation for turbidity.

The use of an in situ nitrate sensor based on UV spectroscopy can help us to monitor environmental changes on a finer spatial and temporal scale, and it has wide prospects for application in freshwater systems and in the monitoring of sewage. The turbidity-compensation method proposed in this study can be used to improve the calibration and data-processing procedures of an in situ nitrate sensor to detect nitrate highly accurately in turbid water. Significantly, in this study, the water samples were prepared by using formazine turbidity particles, which generally conform to a normal distribution with a mean volume diameter of 2.5 µm and may not completely characterize the turbidity of some water bodies [22]. For example, in near-shore estuarine-water environments, in which larger particles are present and uniformity is poorer than in formazine turbidity solutions, the effect of compensation using this method may be affected. In addition, the presence of CDOM in water also affects the detection of nitrate due to its strong absorption in ultraviolet regions. Therefore, additional consideration should be given to eliminating the interference of CDOM to further improve the accuracy with which the concentration of nitrate in water is predicted.

## 3. Materials and Methods

### 3.1. Samples

The experiment was divided into two parts: research and verification of the method. The characteristics of the effect of turbidity on the absorption spectra of nitrate were investigated by single-solution samples of nitrate and turbidity and mixed-solution samples, and a turbidity-compensation model was established on the basis of the difference spectra of the nitrate-solution samples and the mixed-solution samples. The model was further validated with five sets of turbid mixed-solution samples of nitrate at arbitrary concentrations.

A number of samples with different nitrate solutions and levels of turbidity were created to investigate their UV-absorption characteristics at different wavelengths by measuring their absorption spectra, as follows. The data on the nitrate’s absorption spectra were used as a modeling set for the model predicting the nitrate concentration. According to the standards for surface-water environmental quality in China (GB3838-2002), the nitrate-concentration range in the solution samples was set to 0.1–5 mg/L to meet the general requirements of detecting nitrate in surface water. The standard formazine solution is widely used worldwide for the determination of turbidity in water because of its good optical stability. Deionized water, a standard nitrate solution (analytical grade), and the standard formazine solution used for measuring turbidity (analytical grade) were used to prepare the water samples. The deionized water was supplied by a Milli-Q water-purification system (Millipore, Billerica, MA, USA). Nitrate solutions of 0.1, 0.2, 0.5, 1.0, 1.5, 2.0, 2.5, 3.0, 4.0, 5.0 mg/L were obtained by diluting standard nitrate solutions, and turbidity solutions of 1, 5, 10, 15, 20, 30, 40, 50 NTU were obtained by diluting standard solutions of formazine. The nitrate concentration was calculated as the concentration of nitrogen in the solution.

To investigate the effect of turbidity on nitrate’s absorbance spectra and to develop a compensation model, 15 mixed-solution samples with different levels of nitrate and turbidity were prepared. In addition, to validate the compensation method, five different mixtures were produced with random concentrations of nitrate and levels of turbidity. The concentrations used in the samples are shown in Table 3.

### 3.2. Measurements

A UV-vis spectrophotometer (UV-8000s, LASPEC, Shanghai, China) was used to measure the absorption spectra of different solutions from 200 to 400 nm. All the samples were measured in a 1-cm quartz cuvette. Deionized water was used as the reference. The scanning speed was set to medium and the spectral resolution was set to 0.5 nm.

### 3.3. Spectral-Subtraction Method

**Figure 8:**
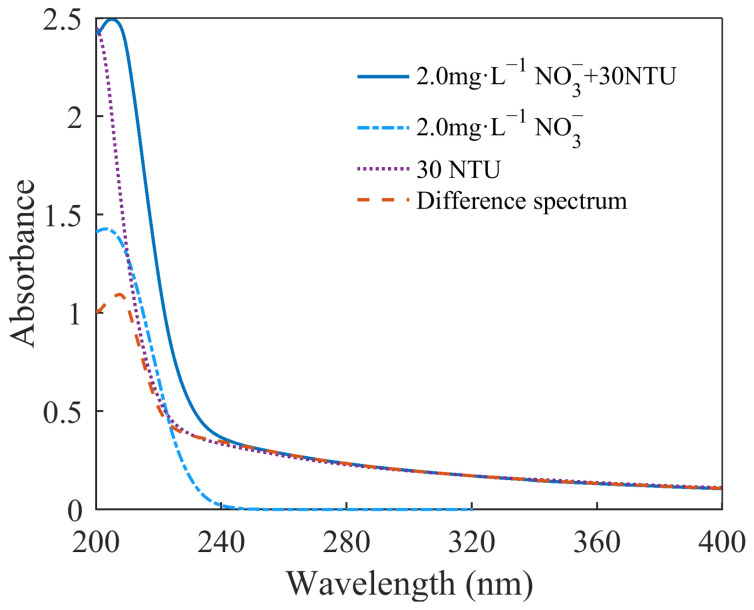
Spectra of the mixed solution, the nitrate solution, the turbidity solution, and their difference spectrum.

The effect of turbidity on the nitrate’s absorption spectra was investigated using a difference spectra obtained by the spectral-subtraction method. An example of a difference spectrum is shown in Figure 8. We acquired the spectrum of mixed nitrate and turbidity solutions, as well as their individual spectra. By subtracting the nitrate spectrum from the spectrum of the mixed solution, the difference spectrum was obtained. The difference spectrum reflects the effect of turbidity on the absorption of nitrate. As can be seen in Figure 1, the appearance of turbidity caused an increase in the total absorbance, but this increase was not equal to the absorbance contributed by the turbidity. This phenomenon is related to molecular interactions in the solution. These are explained in more detail in the Section 2.

### 3.4. Turbidity-Compensation Method

According to the correlation coefficient between the difference spectra and the turbidity, it can be seen that an increase in the absorbance in a specific wavelength range is linearly correlated with the increase in turbidity. The correlation coefficient is calculated in Section 2.1. The change in the absorbance of nitrate as a result of a change in turbidity can be calculated by linear fitting of difference spectra of various levels of turbidity. The spectra of nitrate can then be extracted by subtracting the effect of turbidity from the mixed spectra. The specific steps of the turbidity-compensation method are described below.

Step 1: The absorption spectra of mixed solutions (A_mixture_) and nitrate solutions (A_nitrate_) are measured. The turbidity-prediction model is built through linear regression of the spectral integral at a selected wavelength range and the turbidity value of mixed solutions.

Step 2: The difference spectra of mixed solutions and nitrate solutions are obtained by spectral-subtraction method, which are known as the reference compensation curves (A_ref_). The turbidity values of the difference spectra are known as the reference turbidity values (Tur_ref_). These are calculated as shown in Equations (2) and (3), where n is the number of difference spectra and λ is the wavelength.

(2) $$A_{\mathrm{ref}}=A_{\mathrm{mixture}}-A_{\mathrm{nitrate}}=\left[\begin{array}{ccc} A_{1}^{1} & \cdots & A_{\lambda }^{1}\\ \vdots & \ddots & \vdots \\ A_{1}^{n} & \cdots & A_{\lambda }^{n} \end{array}\right]$$

(3) $${\mathrm{Tur}}_{\mathrm{ref}}=\left[\begin{array}{c} T^{1}\\ \vdots \\ T^{n} \end{array}\right]$$

Step 3: The regression parameters a_i_ (i = 1, 2, …, λ) and b_i_ (i = 1, 2, …, λ) are determined by linear regression for each wavelength using A_ref_ and Tur_ref_, as shown in Equation (4).

(4) $$A_{\mathrm{ref}}{}^{\prime }=\left[\begin{array}{c} a_{1}\\ \vdots \\ a_{\lambda } \end{array}\right]\cdot {\mathrm{Tur}}_{\mathrm{ref}}{}^{\prime }+\left[\begin{array}{c} b_{1}\\ \vdots \\ b_{\lambda } \end{array}\right]$$

Step 4: The absorbance effect of any level of turbidity (A_tur_) can be calculated as shown in Equation (5), where Tur_any_ is the turbidity value of an unknown mixed solution. The turbidity value is calculated by the turbidity-prediction model.

(5) $$A_{\mathrm{tur}}=\left[\begin{array}{c} a_{1}\\ \vdots \\ a_{\lambda } \end{array}\right]\cdot {\mathrm{Tur}}_{\mathrm{any}}+\left[\begin{array}{c} b_{1}\\ \vdots \\ b_{\lambda } \end{array}\right]$$

Step 5: The spectrum of any mixed nitrate and turbidity solution after compensation (A_residual_) is achieved by subtracting A_tur_ from the raw spectrum A_any_, as shown in Equation (6).

(6) $$A_{\mathrm{residual}}=A_{\mathrm{any}}-A_{\mathrm{tur}}$$

### 3.5. Nitrate-Prediction Model

The nitrate-prediction model was constructed by using partial least squares (PLS), a multivariate calibration method. The principle of PLS is to extract the principal components of independent variables and then establish the regression of the dependent variables on these until accurate predictions can be achieved [23]. To improve the model’s accuracy, the modeling-wavelength range was optimized through interval partial least squares (iPLS), which can help us focus on important spectral regions and eliminate interference from other regions [24]. In this method, the measured spectra were evenly divided into different subintervals. All samples were used for PLS modeling in each subinterval, after which the interval with minimum root mean square error of cross-validation (RMSECV) was selected as the optimal wavelength interval.

### 3.6. Process of Calculating the Nitrate Concentration

**Figure 9:**
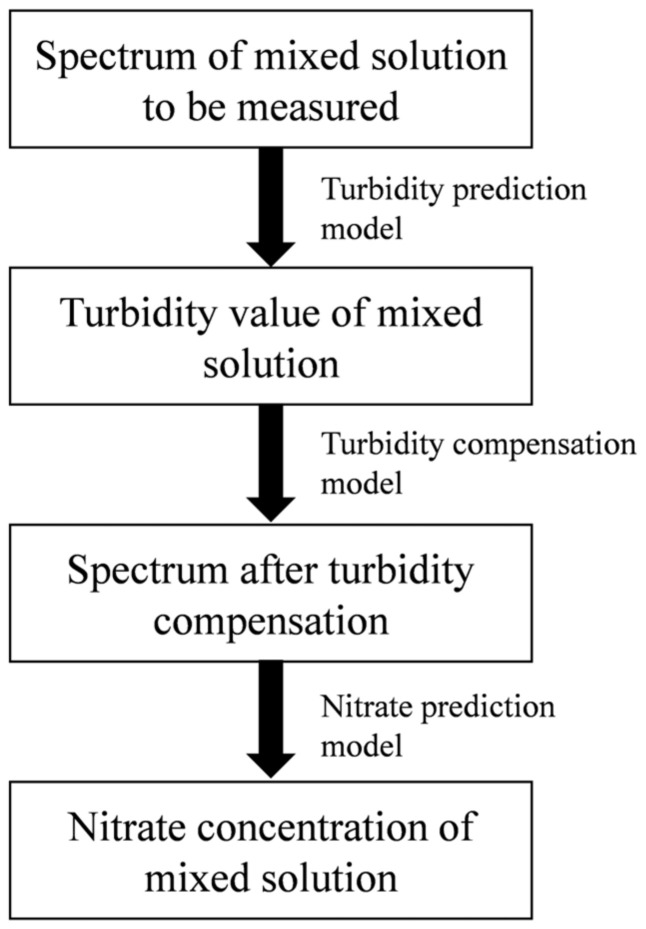
The process for calculating the nitrate concentration.

The process of calculating the nitrate concentration is shown in Figure 9. The turbidity-prediction model, turbidity-compensation model, and nitrate-prediction model were built in advance using the abovementioned methods. Firstly, the absorption spectrum of a mixed solution with an unknown concentration was measured. Secondly, the turbidity of the mixed solution was calculated by using the turbidity-prediction model. Thirdly, the turbidity-compensation model was applied to calculate the turbidity-compensation curve for any value of turbidity. The spectrum after turbidity compensation was obtained by subtracting the turbidity-compensation curve from the raw spectrum. Finally, the absorption spectrum after turbidity compensation was inputted into the nitrate prediction model, and the nitrate concentration was obtained.

### 3.7. Model Validation

The whole model was evaluated according to the root mean square error of prediction (RMSEP), the correlation coefficient (R^2^), and the relative error (RE). These are defined as follows (Equations (7)–(9))

(7) $$\mathrm{RMSEP}=\sqrt{\frac{\sum_{i=1}^{m}{\left({\hat{c}}_{i}-c_{i}\right)}^{2}}{m}}$$

(8) $$R^{2}=1-\frac{\sum_{i=1}^{m}{\left({\hat{c}}_{i}-c_{i}\right)}^{2}}{\sum_{i=1}^{m}{\left(c_{i}-\bar{c}\right)}^{2}}$$

(9) $$\mathrm{RE}=\frac{{\hat{c}}_{i}-c_{i}}{c_{i}}\times 100\%$$

where ${\hat{c}}_{i}$ is the predicted value in the ith sample, $c_{i}$ is the true value in the ith sample, $\bar{c}$ is the mean of the predicted value of all samples, and m is the number of prediction samples.

Leave-one-out cross-validation was used to validate the performance of the PLS models in predicting new data [25]. A total of N-1 elements out of n elements in the whole set were used as the training sets to build models for this analysis, and the remaining element acted as a test set to validate the model. After several rounds of cross-validation, the average validation results were obtained and used to estimate the overall prediction performance of the model. The root mean square error of cross-validation (RMSECV) was the mean value of the RMSEP obtained from the results of several rounds of cross-validation.

## 4. Conclusions

A method of turbidity compensation based on difference spectra was proposed for measuring nitrate. In this study, the two main aims were to (i) study the effect of turbidity on the absorption spectra of nitrate and (ii) to establish a process of compensating for turbidity, including the establishment of a turbidity-prediction model, a turbidity-compensation model, and a nitrate-prediction model. The effectiveness of the compensation method was further verified by experiments. The results showed that the turbidity-compensation method performed well, making the relative error of the predicted nitrate concentration decrease from 50.33% to 1.33%. The following conclusions can be drawn.

(i) The influence of turbidity on nitrate varies with wavelength. The change in absorbance caused by turbidity decreased with the increase in the nitrate concentration at wavelengths of 200–230 nm, and the same level of turbidity had the same effect on the absorbance of nitrate after 230 nm.
(ii) The nitrate-prediction model established in the optimal modeling interval selected by the data analysis of the difference spectra combined with the iPLS algorithm had good predictive accuracy. Meanwhile, arbitrary concentrations of nitrate and levels of turbidity in the water were suitable for the model because of the chosen wavelength.
(iii) The turbidity-compensation method based on the ultraviolet difference spectra can eliminate the interference of turbidity and improve the accuracy with which nitrate is detected in turbid water.
(iv) Although the proposed turbidity-compensation method was effective, there were still limitations in this study, such as the fact that CDOM was not considered when the method was applied to natural water. Further investigations will focus on fully understanding the effects of CDOM and studying the related compensation algorithms, which may improve the accuracy with which nitrate is predicted. Finally, experimental validation will be performed on natural-water samples.

## Figures and Tables

**Table 1:** Regression parameters in the wavelength range of 230–240 nm.

Parameter	The Values in the Wavelength Range of 230–240 nm (the Spectral Interval is 0.5 nm)
$a_{\lambda }$	(0.0127; 0.0125; 0.0124; 0.0123; 0.0122; 0.0121; 0.0120; 0.0119; 0.0118; 0.0117; 0.0116; 0.0115; 0.0114; 0.0114; 0.0113; 0.0112; 0.0111; 0.0111; 0.0110; 0.0109; 0.0109)
$b_{\lambda }$	(0.0333; 0.0327; 0.0322; 0.0317; 0.0311; 0.0305; 0.0303; 0.0298; 0.0293; 0.0289; 0.0285; 0.0280; 0.0277; 0.0275; 0.0276; 0.0275; 0.0272; 0.0268; 0.0267; 0.0265; 0.0263)

**Table 2:** Prediction results of mixed samples before and after turbidity compensation.

Endpoint	Relative Error (100%)	RMSEP (mg L^−1^)	R^2^
Uncompensated	50.33%	0.7845	0.7596
Compensated	1.33%	0.0359	0.9995

**Table 3:** Composition of the mixed-solution samples with varying levels of nitrate and turbidity.

NO.	Nitrate (mg·L^−1^)	Turbidity (NTU)	NO.	Nitrate (mg·L^−1^)	Turbidity (NTU)
1	0.2	10	11	4	10
2	0.2	20	12	4	20
3	0.2	30	13	4	30
4	0.2	40	14	4	40
5	0.2	50	15	4	50
/			Test Samples		
6	2	10	1	0.5	13
7	2	20	2	1	22
8	2	30	3	2	8
9	2	40	4	3	41
10	2	50	5	5	16

## Data Availability

The data presented in this study are available on request from the corresponding author.
